# Burden, Incidence, and Spatial Distribution of Schizophrenia in Ecuador (2010–2021): A Nationwide Hospital Discharge Analysis

**DOI:** 10.3390/ijerph23030310

**Published:** 2026-03-01

**Authors:** Alberto Rodríguez-Lorenzana, Sarah J. Carrington, Marco Coral-Almeida, Diana Álvarez-Mejía, Mabel Torres-Tapia, Guido Mascialino

**Affiliations:** 1 Health Sciences Department, Universidad Pública de Navarra, 31008 Pamplona, Spain; alberto.rodriguez@unavarra.es; 2 Universidad Espíritu Santo, Samborondón 091650, Ecuador; sjcarring@gmail.com; 3 Biochemoinformatics Group, General Direction of Research and Liaison, Veterinary Medicine Department, Universidad de las Américas, Quito 170124, Ecuador; marco.coral@udla.edu.ec; 4 Universidad de Salamanca, 37008 Salamanca, Spain; idu078447@usal.es; 5 Psychology and Education School, Universidad de las Américas, Quito 170137, Ecuador; mabetorres88@gmail.com

**Keywords:** schizophrenia, epidemiology, Ecuador, disease burden

## Abstract

**Highlights:**

**Public health relevance—How does this work relate to a public health issue?**

- This study delivers the first nationwide evaluation of schizophrenia incidence, disease burden, and spatial distribution in Ecuador over an 11-year period.
- The identification of geographic clustering and sex- and age-based diagnostic disparities highlights unequal access to mental health services across the country.

**Public health significance—Why is this work of significance to public health?**

- Schizophrenia accounts for a considerable disease burden (reaching 289.8 DALYs per 100,000 population), exceeding estimates reported in comparable settings and reinforcing its status as a leading contributor to disability.
- A sustained temporal decline in incidence, notably accentuated during the COVID-19 period, reflects shifts in healthcare utilization and potential system-level barriers to psychiatric care.

**Public health implications—What are the key implications or messages for practitioners, policy makers and/or researchers in public health?**

- Findings underline the need to strengthen mental health infrastructure, expand early detection strategies, and reduce stigma to address the treatment gap and resulting disability burden.
- Spatial concentration of cases provides an evidence-based foundation for strategic resource allocation, informing targeted interventions and region-specific mental health policy planning.

**Abstract:**

Schizophrenia is a chronic mental disorder affecting approximately 1% of the global population and imposing a significant economic and social burden. In Ecuador, comprehensive data on its incidence, burden, and spatial distribution are scarce. This study aims to estimate the hospital-diagnosed incidence, disease burden, and spatial patterns of schizophrenia in Ecuador using national hospital discharge records from 2010 to 2021. A retrospective observational study was conducted using publicly available hospital discharge records from the Instituto Nacional de Estadística y Censos (INEC). Schizophrenia cases were identified using ICD-10 codes F20–F29. Incidence rates per 100,000 population were estimated with 95% Poisson confidence intervals. Disability-Adjusted Life Years (DALYs) were calculated under three scenarios: no discounting or age weighting, 3%-time discounting, and both. Spatial clusters were identified using SATSCAN software v10.1.2. A total of 10,542 schizophrenia cases were recorded between 2010 and 2021, with an overall incidence rate of 5.36 per 100,000 population (95% CI: 5.26–5.46). Incidence significantly decreased over time (*p* = 0.029). The estimated burden ranged from 153.05 to 289.78 DALYs per 100,000. High-incidence clusters were identified in Guayas and Pichincha provinces. This study provides the first nationwide assessment of schizophrenia in Ecuador, offering critical insights for health policy development, resource allocation, and improved care strategies.

## 1. Introduction

Schizophrenia is a profound and persistent mental disorder that significantly impairs the functioning of affected individuals [1]. While its clinical manifestation typically occurs during late adolescence or early adulthood, schizophrenia is widely conceptualized as a neurodevelopmental disorder with origins in early brain development [2]. Diagnosis typically follows the first psychotic episode, but even in the prodromal phase, patients often exhibit milder symptoms that correlate with cognitive and social impairments [3]. Without appropriate treatment, affected individuals face enduring functional challenges [4], often marked by incomplete remissions, a predominance of negative symptoms, prolonged hospital stays, and heightened risk for co-occurring substance abuse disorders and depression [5]. The disease’s trajectory and severity vary widely among patients [6].

Globally, over 24 million individuals suffer from schizophrenia [7], maintaining a consistent prevalence of around 1% [8]. While the prevalence may seem low relative to other psychiatric conditions, the disorder’s magnitude, both for the individual and society, is vast. The condition impacts all aspects of life, including employment prospects, broader health, and social welfare [3]. Importantly, schizophrenia represents one of the leading contributors to disability among mental disorders, accounting for a disproportionate share of years lived with disability worldwide [9,10].

This substantial disability burden, particularly during acute and chronic phases of the illness, also translates into indirect economic consequences, including productivity losses, prolonged hospitalizations, and increased healthcare utilization [11]. However, the primary global impact of schizophrenia is reflected in its contribution to disability-adjusted life years (DALYs), driven predominantly by years lived with disability rather than premature mortality [9]. Indeed, schizophrenia ranks as one of the leading causes for years lived with disability, marking it as a severe mental illness [10]. The disparity in life expectancy between general and clinical populations stands at a striking 15 to 20 years. This is in part due to an elevated risk of cardiovascular diseases and suicide accompanying a schizophrenia diagnosis [3,12]. Moreover, these demographics face additional risks including metabolic syndrome, diabetes, sedentary lifestyles, and smoking. Often, they lack preventive care, partly attributable to inadequate health education and pervasive stigmatization [13]. The diagnosis further limits their access to regular medical care. All of these factors contribute to the noted reduction in life expectancy [12].

In Latin America, there is a notable paucity of research addressing the epidemiology and disease burden of schizophrenia. A 2020 study conducted in Brazil looking at schizophrenia and other common mental disorders reported an incidence rate in 2009 of 77.44 per 100,000 inhabitants. This translated into an annual expenditure of about 67 million dollars and an average hospitalization period of 45 days [14]. Epidemiological studies compiled by the Pan American Health Organization indicate the existence of research addressing prevalence estimates; however, they do not consistently report annual population-based incidence rates, confirming a persistent data gap across many Latin American countries [15]. In terms of prevalence, estimates derived from the Global Burden of Disease 2019 study indicate relatively homogeneous age-standardized rates across most Latin American subregions, ranging from approximately 270 to 280 cases per 100,000 population. Southern Latin America, which includes countries such as Argentina, Chile, and Uruguay, shows the highest prevalence in the region (313.4 per 100,000), while Andean, Central, and Tropical Latin America present slightly lower but largely overlapping estimates [9]. The considerable differences in estimates per region may be due to differences in study design, quality, dates, and other variables.

Recent data from PAHO [16] and the World Health Organization (WHO) [7] suggest that in Ecuador, mental health issues affect 30 out of every 100 individuals, a prevalence akin to that observed in countries like the United States. Among these mental health conditions, schizophrenia has the highest case prevalence (39%) in psychiatric hospitals nationwide, followed by affective disorders (26%) [17].

In Ecuador, mental health care for schizophrenia is delivered across outpatient and inpatient services; however, hospital admission is typically required for acute episodes, severe symptom exacerbations, or situations involving significant functional impairment or risk. Consequently, hospital discharge records capture all hospitalized cases of schizophrenia, but do not represent the full spectrum of cases managed exclusively in outpatient or community-based settings.

As of now, Ecuador lacks a dedicated epidemiological study on schizophrenia, rendering the precise economic implications of the disease elusive. It is important to note that epidemiological estimates based on hospital discharge records reflect diagnosed cases within the healthcare system and may underestimate the true population incidence of schizophrenia, particularly among individuals who have never been hospitalized. This study endeavors to bridge this gap, delving into data concerning individuals with schizophrenia admitted to the country’s health institutions. The research aims to estimate the hospital-diagnosed incidence, disease burden, and spatial patterns of schizophrenia in Ecuador using national hospital discharge records from 2010 to 2021. Ultimately, by crafting and disseminating this study, we hope to bolster informed decision-making processes related to the promotion, prevention, and treatment of schizophrenia.

## 2. Methods

A review was conducted using a publicly available database by the Instituto Nacional de Estadística y Censos (INEC), which compiles national statistics, including hospital data from all over Ecuador. The database contains anonymized information on hospital discharge diagnoses and demographic details from public and private health facilities between 2010 and 2021. Accordingly, the incidence estimates reported in this study correspond to hospital-diagnosed schizophrenia, defined as new recorded hospital admissions with an ICD-10 diagnosis of schizophrenia during the study period. Diagnoses were classified using the International Classification of Diseases (ICD-10). Diagnoses related to schizophrenia were identified with ICD-10 codes F20, which include the categories: F20.0 paranoid schizophrenia; F20.1 hebephrenic schizophrenia; F20.2 catatonic schizophrenia; F20. 3 undifferentiated schizophrenia; F20.4 post-schizophrenic depression; F20.5 residual schizophrenia; F20.6 simple schizophrenia; F20.8 other schizophrenia; and F20.9 schizophrenia, unspecified. Ethnic or racial origin was reported, including Indigenous, Afro descendant, Mestizo, Montubio, and White. Data included those who did not report on their race or ethnicity.

### 2.1. Sources of Information

In Ecuador, the population was estimated to be 17,751,277 in 2021, with the 2010 census recording 15,012,228 inhabitants [18]. The study used data from 2010 to 2021 from the national records of hospital deaths and admissions [19,20]. These data were derived from the INEC hospital discharge database, which is anonymized and does not include a unique patient identifier. Consequently, repeated hospitalizations of the same individual could not be identified or excluded, and each hospital’s discharge was treated as an independent event. These records, published by INEC, were processed using Microsoft Excel and analyzed using the DALY calculator in R v4.1.2 [21]. ICD-10 codes F20-F29 were used to identify schizophrenia-related outcomes reported.

### 2.2. Estimation of the Burden of Disease

To assess the impact of schizophrenia, Disability Adjusted Life Years (DALYs) were calculated. DALYs combine years lived with disability (YLDs) and years of life lost (YLLs) due to premature mortality. The DALYs were determined by multiplying the number of schizophrenia-related deaths by the remaining life expectancy at the age of death, calculated using the R program with GBD 2010 data, assuming a life expectancy of 86.02 years. Three approaches were used to calculate the DALYs: no time discounting or age weighting, 3% time discounting, and both, following the methodology of [22].

Hospital admission data represent only a portion of the schizophrenic population with access to medical care; therefore, they are not suitable for estimating the total DALYs at the national level, but rather for estimating DALYs evidenced through the healthcare system. Due to the lack of data on the severity of illness in patients, an average disability weight (PD) of 0.714 was used, based on the weighted average of acute and chronic schizophrenia, derived from the studies by [23,24]. Age-specific data showed that the incidence of schizophrenia diagnosis varies across age groups, with higher rates in men aged 15–44 years (89 per 100,000) and women aged 45–60 years (97 per 100,000).

### 2.3. Statistical Analyses

Descriptive statistics were collected on year and sex variables, and Pearson’s chi-square, Poisson regressions, and linear regressions were used to assess associations of incidence with annual evolution and sex, with significance determined at *p*-values < 0.05. Incidences were reported with Poisson 95% confidence intervals. All analyses and figures were generated in R v4.1.2 [21].

### 2.4. Spatial Analyses and Statistical Methods

Spatial analysis was performed to identify geographic clusters with higher incidence rates of schizophrenia, distributing cases by canton according to the patients’ habitual residence as recorded in the INEC hospital discharge database, rather than the location of the reporting hospital. Spatial clusters were identified using Kulldorff’s purely spatial scan statistic implemented in SaTScan^TM^ v10.1.2 [25], following the methodologies of Ron-Garrido et al. [26] and Kulldorff [27], with a purely spatial approach. A discrete Poisson model was applied to compare the observed number of schizophrenia cases in each canton with the expected number under a spatial randomness assumption, using population counts as offsets. Circular scanning windows of variable radius were used to detect areas of significantly elevated risk. Statistical significance of spatial clusters was evaluated using likelihood ratio tests and Monte Carlo hypothesis testing with 999 random replications, with significance set at *p* < 0.05. The likelihood ratio test was used to identify the most likely and secondary clusters, and the Gini coefficient was applied to support cluster selection. Final spatial analyses were performed using QGIS v3.8 Zanzibar, and maps were created by the authors using official INEC shapefiles.

INEC population projections, which vary annually, were used to adjust incidence rates during the study period. Incidence rates were reported in absolute numbers and relative rates per 100,000 population, with 95% Poisson confidence intervals calculated using the epitools package in R v4.1.3 [21].

## 3. Results

From 2010 to 2021, a total of 10542 schizophrenia cases were reported where 5779 were male and 4763 were female. The highest incidence rate was recorded in 2010 while the lowest was registered in 2020 (Table 1). There was a negative association between time and incidence rates (*p*-value = 0.0293). There was a difference between males and females in the occurrence of schizophrenia.

### 3.1. Characteristics by Gender

During the study period, 4763 female schizophrenia cases were registered. The highest incidence rate was in 2011, and the lowest in 2020 (Supplementary Table S2). A negative association between time and incidence rates was found (*p*-value = 0.0127), suggesting a notable decrease in cases between 2010 and 2021. For males, 5779 schizophrenia cases were identified. The highest rate occurred in 2011, and the lowest in 2013 (Supplementary Table S3). A negative association was also observed (*p*-value = 0.112), indicating a decrease in cases over the same period.

### 3.2. Characteristics by Age of Onset and Sex

**Figure 1:**
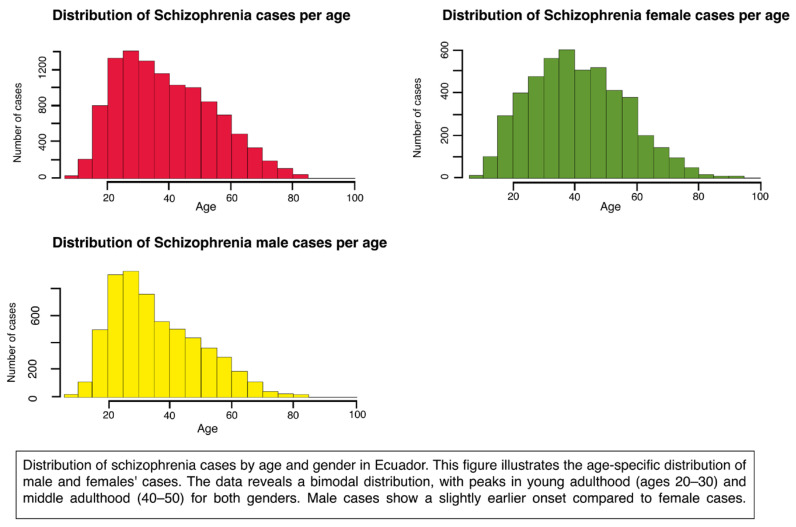
Distribution of schizophrenia total (red), male (yellow) and female (green) cases per age.

Further analyses compared the age at diagnosis and/or treatment for schizophrenia by sex (Figure 1). The mean age was 38.82 with confidence intervals at 95% [38.53; 39.11]. The mean age of diagnosis/treatment for females was 41.24 with confidence intervals at 95% [40.81; 41.68]. For males, the mean age of diagnosis was 36.82 with confidence intervals at 95% [36.44; 37.20]

A comparison between males and females was made. There was a positive association, proposing a notable difference between the ages of diagnosis (*p*-value < 0.000001). Male patients were diagnosed/treated at a younger age than female patients.

### 3.3. Burden of Disease Expressed in DALYs

The estimated burden of disease of schizophrenia in Ecuador (2010–2021) varied from 153.054 to 289.785 per 100,000 population on average depending on the scenario used for estimation (discount rates and age weighting scenarios, as above escribed; Table S1). Hospital attention in the public sector contributed with the highest average proportion of DALYs per sector. Hospital care in the private sector was the group with the lowest DALY average contribution. The results are detailed in Table 2.

It is important to emphasize that the DALY estimates presented in this study were derived exclusively from hospital discharge and death records and therefore represent proxy indicators of hospital-based fatal burden rather than the total burden of schizophrenia in the Ecuadorian population. Because hospital datasets lack information on disease severity, duration, and long-term disability, years lived with disability (YLDs) could not be reliably estimated. Consequently, all DALYs in this analysis were driven exclusively by years of life lost (YLLs) attributed to hospital-registered deaths coded as schizophrenia-related, a phenomenon that is rare and potentially subject to misclassification.

Accordingly, YLLs accounted for 100% of the total DALY estimates, while YLDs contributed 0% across all sectors analyzed.

### 3.4. Spatial Distribution of Schizophrenia in Ecuador

In the spatial cluster map (Supplementary Figure S1), the distribution of schizophrenia incidence case clusters is highlighted using a cluster analysis approach. The clusters are identified by red dots, each dot representing the center of a canton with a significant cluster for schizophrenia incidence cases. Conversely, regions with no significant clusters are represented in plain white. 

In the map (Supplementary Figure S1), the spatial distribution of the incidence of schizophrenia is visualized. Darker blue shades indicate higher incidences of schizophrenia hospitalized cases, while lighter areas correspond to lower incidences of schizophrenia hospitalized cases. Thicker black lines represent province borders, while light grey lines represent cantonal borders. Significant clusters are presented in Supplementary Table S4.

## 4. Discussion

This study represents the first comprehensive analysis of the incidence and disease burden of schizophrenia in Ecuador based on national hospital records. The incidence estimates reported in this study should be interpreted as hospital-diagnosed incidence and therefore reflect patterns of access to and utilization of inpatient psychiatric services, rather than the true population incidence of schizophrenia. Between 2010 and 2021, 10,542 cases were identified. There was a consistent downward trend in schizophrenia incidence, decreasing from 7.24 cases per 100,000 individuals in 2010 to 5.10 cases per 100,000 in 2021. These findings align with the 2017 Global Burden of Disease (GBD) report, which also showed a decline in the global age-adjusted incidence rate (ASIR) from 1990 to 2017. However, other research highlights a global increase in diagnoses and treatment of schizophrenia. For example, global prevalence increased from 13.1 million in 1990 to 20.9 million in 2016 [23], and a study from China recorded a rise of 4.07 million cases between 1990 and 2010 [28]. Richter et al. [29] suggest that this increase in diagnoses is due to sociodemographic shifts, while Shivashankar et al. [30] attribute it to longer life expectancy, improved diagnostic capabilities, and reduced stigma.

In Latin America, the establishment and growth of programs dedicated to the diagnosis, prevention, care, and rehabilitation of mental disorders remain nascent. Such underdevelopment might substantially influence the way previously mentioned sociodemographic factors manifest in the region. Notably, there exists a staggering treatment gap for schizophrenia patients in Latin America, with 56.4% of patients going untreated [31]. Such an alarming scenario can be partially attributed to the region’s governments disproportionately underinvesting in mental health as compared to other public health challenges. This is possibly rooted in cultural biases that de-prioritize mental health amongst the population as well as amongst health professionals (i.e., not trained to recognize or work with) and services (e.g., most health insurance do not recognize treatment for mental health conditions as a claimable expense). Consequently, public service resources are stretched thin, leading to compromises in the quality and accessibility of diagnostic and therapeutic services [32]. The incidence rates unearthed in our study for Ecuador echo this broader trend, aligning closely with the Latin American average of 4.4 per 10,000 inhabitants, as documented by Kohn [31].

In 2020, there was a drop in schizophrenia diagnoses across both public and private sectors, likely due to the COVID-19 pandemic. This decline should be interpreted with caution, as it likely reflects reduced access to and utilization of mental health services during the pandemic rather than a true decrease in the underlying occurrence of schizophrenia. Health systems redirected resources towards COVID-19 prevention and treatment, potentially reducing care for other mental health issues [33]. The pandemic also worsened existing inequities in access to mental health services [34], and fear of viral transmission may have deterred individuals from seeking in-hospital psychiatric care [35].

Interestingly, our findings differ from those reported in other countries [36,37], which highlight an increase in schizophrenia diagnoses during COVID-19. These studies highlight several pandemic-induced stressors (unemployment, loss of loved ones, and social isolation) as possible contributors to the deterioration of psychological well-being. A distinguishing factor between Ecuador and the countries studied in these reports is the accessibility and fiscal priority of public health services, and the social stigma related to mental health treatment. These disparities could have generated a greater reluctance of individuals to seek hospital services during a period when the health system was stretched to its limits.

Our results also reveal clear epidemiological disparities between sexes. A higher incidence was observed in men (5779 cases, incidence of 5.93) compared to women (4763 cases, incidence of 4.80). This is consistent with other research identifying differences in the manifestation of schizophrenia between males and females [38], with variations in disease progression [39], neurodevelopmental processes [40], and debut [41]. However, while global studies consistently report a higher number of diagnoses in men, they also agree that prevalence rates between sexes do not differ significantly [42,43].

In Ecuador, the average age at diagnosis of schizophrenia is 36.82 years for men and 41.24 years for women, both higher than global averages. Importantly, the age of onset of schizophrenia (usually occurring during late adolescence or early adulthood) differs conceptually from the age at diagnosis, which reflects the timing of clinical recognition within the healthcare system.

Therefore, higher ages at diagnosis should not be interpreted as late onset of the disorder, but rather as potential indicators of diagnostic delay. The age of onset and diagnosis of schizophrenia can be influenced by social, economic, and political factors.

For example, in Honduras, the average age of onset is 21.4 years, with diagnosis at 23.7 years [44]. In Colombia, hospital-based studies have reported mean ages close to 40 years at first contact with mental health services, rather than true age of onset, suggesting delayed diagnosis rather than late emergence of schizophrenia [45]. In Spain, data from a large clinical cohort indicate a mean age of 37.6 years at first assessment and 39.3 years at first diagnosis [46]. These figures reflect patterns of service utilization in clinical settings and should not be interpreted as delayed onset, as early detection and access to specialized mental health care are generally more established in high-income countries. In Ecuador, several factors could explain the higher age of diagnosis, including possible underreporting or underestimation of cases, limited mental health prioritization, organizational challenges, and widespread stigma. Such barriers to early detection and timely care are characteristic of many low- and middle-income countries, where under-resourced mental health systems, limited trained personnel, and pervasive stigma contribute to delays in diagnosis and treatment of schizophrenia and other severe mental illnesses [47].

Recent findings from the Global Burden of Disease (GBD) 2023 study provide important context for interpreting our results. While substantial global progress has been made in reducing the burden of infectious diseases, mental disorders remain a mounting challenge worldwide, primarily driven by long-term disability rather than premature mortality. The GBD 2023 highlights that increases in healthy years lost are largely attributable to chronic non-communicable conditions, including mental disorders, whose burden is predominantly expressed through years lived with disability. In this context, the declining trend in hospital-diagnosed incidence observed in Ecuador should not be interpreted as a reduction in the true burden of schizophrenia, but rather as a reflection of health system dynamics, barriers to access, and the reliance on hospital-based data sources. Consistent with GBD 2023 findings, regions with constrained mental health services may substantially underestimate the population burden of severe mental disorders, as much of the disability occurs outside inpatient settings and is not captured by mortality or discharge records [48].

Regarding Disability-Adjusted Life Years (DALYs), the data indicate that from 2010 to 2021, there was a disease burden of 289.78 per 100,000 inhabitants in Ecuador. Notably, much more treatment was funneled through the public sector (175.01), in comparison to the private sector (115.06). This could reflect the socio-economic status of patients (or their ability to access other services), or the lack of services in private health (or their relative cost). For a global perspective, He et al. [49] reported that in 2017, schizophrenia accounted for 12.66 million DALYs worldwide. Interestingly, the same study revealed higher incidences in males, with a figure of 6.51 million (95% UI = 4.86 to 8.02 million), compared to females, at 6.14 million (95% UI = 4.61 to 7.54 million). Additionally, Matos et al. [50] underscored that schizophrenia stands as one of the primary causes of DALYs among mental disorders, contributing to 7.4% of the total DALYs globally between 1990 and 2010. Zooming in on South America, in a study from Colombia spanning 2006 to 2012, available data indicated that schizophrenia represented 2.64 DALYs per 1000 inhabitants [51]. These results are comparable to our study.

Although schizophrenia might not be the most prevalent mental disorder, its impact on disease burden cannot be understated. Several factors contribute to the heightened disease burden of schizophrenia: population aging, early onset, low remission rates, and the profound disability often associated with the disorder. This burden is particularly pronounced in low to middle-income countries, Ecuador included.

In terms of the geographic distribution of the incidence of schizophrenia, the provinces of Ecuador with the most registered cases are Guayas, Pichincha, Los Rios, Esmeraldas, Morona Santiago, Chimborazo, Galapagos and Loja. There is a coincidence between the reported incidences and the clusters reflected in Figure S1, which suggests that the identification of clusters could be a useful strategy for the identification of problems and the design of possible response measures. Finally, provincial capitals exhibit a pronounced incidence rate. Because spatial analyses were based on patients’ canton of habitual residence rather than the location of referral hospitals, this observed clustering cannot be attributed to the concentration of specialized health facilities in provincial capitals. Instead, the identified spatial patterns reflect the geographic origin of hospitalized individuals, suggesting that higher incidence rates in provincial capitals are not merely an artifact of patient referral for treatment, even though individuals from more remote areas may travel to these cities due to limited mental health services in their home locales.

### Limitations

This study has limitations tied to the data source and its representativeness. Although information was obtained from both public and private healthcare sectors, the consistency and accuracy of diagnostic recording across these systems cannot be fully assured, as previous literature indicates substantial variability in the quality of hospital records between institutions. Consequently, some degree of misclassification or coding inaccuracy may be present. In addition, because the INEC hospital discharge database is anonymized and lacks a unique patient identifier, repeated hospitalizations of the same individual could not be identified or excluded, potentially resulting in duplicate entries.

Furthermore, as this analysis was based exclusively on hospital discharge and mortality records, schizophrenia is likely underreported in Ecuador, since individuals who were never hospitalized or who received care solely in outpatient or community-based settings were not captured. Relatedly, the estimation of disability-adjusted life years (DALYs) in this study was restricted to hospital-based fatal events and does not reflect the substantial disability burden typically associated with schizophrenia. Unlike population-based burden of disease studies, in which more than 90% of DALYs are driven by years lived with disability (YLDs), the present approach relies on hospital discharge and mortality registries that do not contain information on symptom severity, disease duration, functional impairment, or outpatient care. Therefore, the DALY estimates reported here should be interpreted as proxy indicators of fatal outcomes within the hospital system rather than as measures of the total burden of schizophrenia in the Ecuadorian population.

Finally, the spatial cluster detection performed using scan statistics may be sensitive to methodological choices such as the specification of the maximum spatial window size and other parameter settings. Because sensitivity analyses using alternative window sizes were not conducted, the robustness of the identified clusters to variations in these parameters cannot be formally assessed.

The results of this study should also be interpreted within the sociocultural and health-system context of Ecuador and the region. Healthcare in the country had an increased budget from 2010–2019, with a concomitant increase in the hiring of health personnel [52]. However, more recent data suggest that hospitals have limited resources, particularly medicine and hospital beds, which became apparent during the COVID-19 pandemic [53]. Access to care also remains limited, particularly in rural and low-resource settings, as well as for vulnerable populations such as ethnic minorities and people/regions with low SES [53,54]. These structural constraints may contribute to delayed diagnosis and hospitalizations, which may disproportionately affect younger males, who tend to present with earlier onset and more overt symptomatology. Additionally, persistent stigma surrounding severe mental illness may also influence care seeking [55]. Furthermore, perceptions of mental health needs and legitimacy can vary by gender, thus impacting differences in hospitalization in that variable [56]. Together, these factors suggest that the epidemiological patterns identified in this study reflect not only underlying disease distribution, but also sociocultural norms and systemic barriers shaping access to psychiatric care in Ecuador.

## 5. Conclusions

This is the first study to characterize in detail the incidence and burden of schizophrenia in Ecuador over a period of 11 years. The findings show that, although the incidence rate exhibits lower values compared to the latest estimates worldwide, the burden of the disease exceeds that reported in other countries in the same geographic and socioeconomic demarcation. In addition, a gradual decline in incidence was observed throughout the period investigated, consistent with the results of some previous reports. The results obtained could contribute to the refinement and formulation of health policies, the allocation of government resources, and the improvement of health surveillance systems to improve the quality and scope of care provided to individuals affected by this disorder.

## Figures and Tables

**Table 1:** Cases of schizophrenia from 2010 to 2021.

Year	Number of Cases	Persons-Time at Risk	Incidence Rate in 100,000 Person per Year	Poisson Confidence Intervals at 95%
2010	1095	15,012,228	7.29	[6.87; 7.74]
2011	1037	15,266,431	6.79	[6.39; 7.22]
2012	955	15,520,973	6.15	[5.77; 6.56]
2013	656	15,774,749	4.16	[3.85; 4.49]
2014	836	16,027,466	5.22	[4.87; 5.58]
2015	834	16,278,844	5.12	[4.78; 5.48]
2016	755	16,528,730	4.57	[4.25; 4.91]
2017	881	16,776,977	5.25	[4.91; 5.61]
2018	963	17,023,408	5.66	[5.31; 6.03]
2019	1004	17,267,986	5.81	[5.46; 6.19]
2020	725	17,510,643	4.14	[3.84; 4.45]
2021	801	17,751,277	4.51	[4.21; 4.84]
Total	10,542			
Yearly Mean Incidence			5.36	[5.26; 5.46]

**Table 2:** Hospital-Based Burden Indicators (DALY Proxy Estimates).

	General	Public Sector	Private Sector
**DALY/POP/100,000**			
No age weighting and no discount rate	289.78	175.01	115.07
Age weighting and no discount rate	263.44	159.74	92.52
Age weighting and 3% discount rate	153.05	103.97	60.68
**Contributions of YLD and YLL for DALYs**			
YLD/DALY	100%	100%	100%
YLL/DALY	0%	0%	0%

## Data Availability

The data that support the findings of this study are publicly available and can be accessed at the following link: https://anda.inec.gob.ec/anda/index.php/catalog (accessed on 15 December 2022). These databases are freely accessible and were used in accordance with the terms and conditions specified by the respective sources. No proprietary or restricted data were used in this study.

## Supplementary Materials

The following supporting information can be downloaded at: https://www.mdpi.com/article/10.3390/ijerph23030310/s1. Table S1. Parameters for DALY calculation, Table S2. Female cases of Schizophrenia and incidence by year, Table S3. Male cases of Schizophrenia and incidence by year, Table S4. Significant clusters for Schizophrenia in Ecuador, Figure S1. Spatial distribution and incidence of Schizophrenia clusters in Ecuador (2010–2021).
