# Supplementary material for: Burden, Incidence, and Spatial Distribution of Schizophrenia in Ecuador (2010–2021): A Nationwide Hospital Discharge Analysis
Source: Int J Environ Res Public Health. 2026 Mar 1;23(3):310. doi: 10.3390/ijerph23030310 (PMC13026791; doi:10.3390/ijerph23030310)
Supplement: Supplementary file 1 [file ijerph-23-00310-s001.zip › ijerph-4061851-supplementary.pdf]

Supplementary Table S1.

Parameters for DALY calculation S1

Parameter	Probability distribution	Value range		Source
Population	Fixed by age and sex	15.0 in 2010 – 17.8 million in 2021 inhabitants		INEC [18]
Prevalence of Schizophrenia	Fixed	0 to 97 per 100,000 people		Calculated from data consolidated by INEC [19,20]
Disability Weight: Weighted average of chronic and acute diagnoses (37% and 63%, respectively).	Fixed	Average DW = 0.714		Calculated from data taken from Ferrari et al. [24], and Charlson et al. [23]
Assumed average duration of disability in years (males and females).	Fixed	1 year		Assumed due to unknown duration of illness.
Treatment proportion of male and female	Fixed	1 [range 0 – 1].		Due to the data being used relate entirely to treated cases.
**INCIDENCE BY AGE GROUP (per 100,000 in total population**		**Male**	**Female**	
Age group 0 – 4 years	Fixed	0	0	Calculated from data consolidated by INEC [19,20]
Age group 5 – 14 years	Fixed	2	3	Calculated from data consolidated by INEC [19,20]
Age group 15 – 44 years	Fixed	89	61	Calculated from data consolidated by INEC [19,20]
Age group 45 – 59 years	Fixed	86	97	Calculated from data consolidated by INEC [19,20]
Age group 60+ years	Fixed	48	56	Calculated from data consolidated by INEC [19,20]

Supplementary Table S2.

Female cases of Schizophrenia and incidence by year S2.

Year	Number of cases	Persons-time at risk	Incidence rate in 100,000 person-years	Poisson confidence intervals at 95%
2010	556	7568353	7.35	[6.75;7.98]
2011	485	7698755	6.30	[5.75;6.89]
2012	472	7829061	6.03	[5.50;6.60]
2013	290	7958814	3.64	[3.24;4.09]
2014	377	8087914	4.66	[4.20;5.16]
2015	399	8216234	4.86	[4.39;5.36]
2016	305	8343760	3.66	[3.26;4.09]
2017	358	8470420	4.23	[3.80;4.69]
2018	435	8596147	5.06	[4.60;5.56]
2019	441	8720919	5.06	[4.60;5.55]
2020	296	8844706	3.35	[2.98;3.75]
2021	349	8978739	3.89	[3.49;4.32]
**Total**	4763			
**Yearly Mean Incidence**			4.80	[4.66;4.93]

Supplementary Table S3.

Male cases of Schizophrenia and incidence by year S3.

Year	Number of cases	Persons-time at risk	Incidence rate in 100,000 person-years	Poisson confidence intervals at 95%
2010	539	7443875	7.24	[6.64;7.88]
2011	552	7567676	7.29	[6.70;7.93]
2012	483	7691912	6.28	[5.73;6.87]
2013	366	7815935	4.68	[4.22;5.19]
2014	459	7939552	5.78	[5.26;6.34]
2015	435	8062610	5.40	[4.90;5.93]
2016	450	8184970	5.50	[5.00;6.03]
2017	523	8306557	6.30	[5.77;6.86]
2018	528	8427261	6.27	[5.74;6.82]
2019	563	8547067	6.59	[6.05;7.15]
2020	429	8665937	4.95	[4.49;5.44]
2021	452	8870400	5.10	[4.64;5.59]
**Total**	5779			
**Yearly Mean Incidence**			5.93	[5.77;6.08]

Supplementary Table S4.

Significant clusters for Schizophrenia in Ecuador S4

Cluster	Province(s) Location	Radius in Km	Relative Risk	*p*-value
1	Guayas	Single spot	2.48	< 0.0001
2	Pichincha	Single spot	1.40	< 0.0001
3	Los Ríos	Single spot	2.43	< 0.0001
4	Esmeraldas	Single spot	1.66	< 0.0001
5	Morona Santiago	Single spot	3.42	< 0.0001
6	Chimborazo	Single spot	1.49	< 0.0001
7	Galápagos	Single spot	3.80	0.00049
8	Loja	21.96	2.64	0.027

Supplementary Figure S1. Spatial distribution and incidence of Schizophrenia clusters in Ecuador (2010-2021)

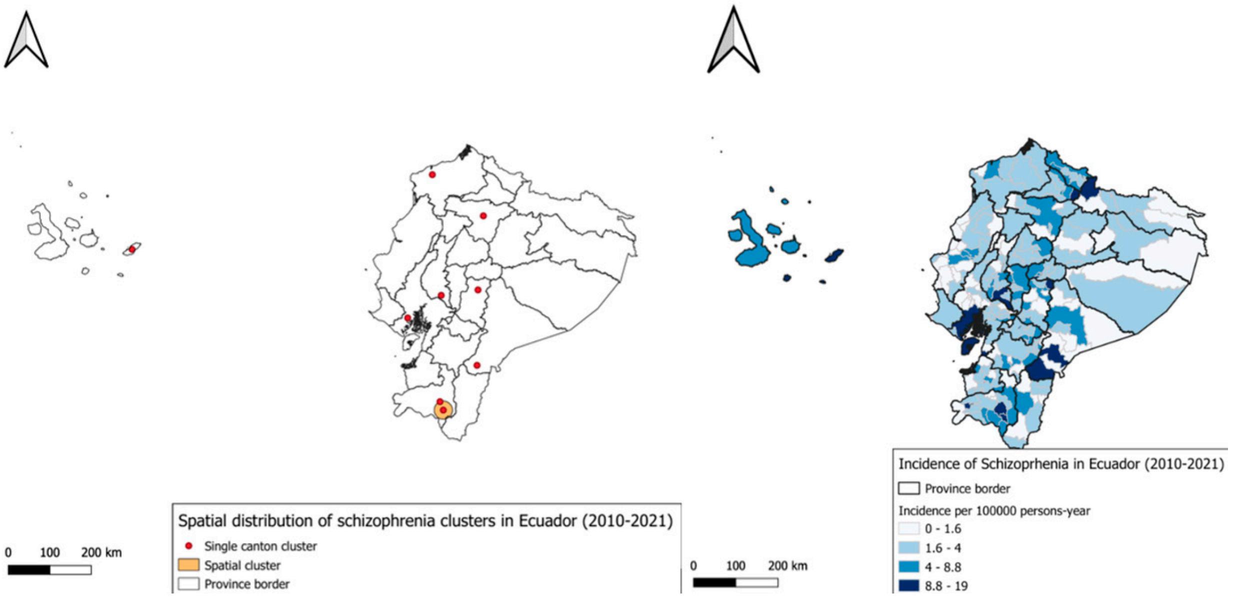

This figure maps significant clusters of schizophrenia incidence and notable regional variations identified using SaTScan spatial analysis. Higher incidences are concentrated in urbanized provinces such as Pichincha and Guayas compared to other rural areas.

18. Instituto Nacional de Estadística y Censos (INEC). Censo de Población y Vivienda. Ecuador en Cifras. Available online: https://www.ecuadorencifras.gob.ec/censo-de-poblacion-y-vivienda/ (accessed on 11 February 2023).
19. Instituto Nacional de Estadística y Censos (INEC). *Camas y Egresos Hospitalarios*. Ecuador: INEC; 2023. Available online: https://www.ecuadorencifras.gob.ec/camas-y-egresos-hospitalarios-2023/ (accessed on 26 February 2023).
20. Instituto Nacional de Estadística y Censos (INEC). *ANDA (National Data Archive)*. Ecuador: INEC; 2023. Available online: https://anda.inec.gob.ec/anda/index.php/catalog#_r=&collection=&country=&dtype=&from=2007

&page=20&ps=&sk=&sort_by=titl&sort_order=&to=2026&topic=&view=s&vk= (accessed on 26 February 2023).

23. Charlson, F.; Ferrari, A.; Santomauro, D.; Diminic, S.; Stockings, E.; Scott, J.; McGrath, J.; Whiteford, H. Global epidemiology and burden of schizophrenia: Findings from the global burden of disease study 2016. *Schizophr. Bull.* **2018**, 44, 1195–1203. https://doi.org/10.1093/schbul/sby058.
24. Ferrari AJ, Saha S, McGrath JJ, Norman R, Baxter AJ, Vos T, Whiteford HA. Health states for schizophrenia and bipolar disorder within the Global Burden of Disease 2010 Study. Popul Health Metr. 2012;10:16.
